# International Retrovirology Association brings together scientists and clinicians to bridge discoveries about human T-lymphotropic viruses from the laboratory to clinical trials

**DOI:** 10.1186/1742-4690-2-22

**Published:** 2005-03-29

**Authors:** Edward Murphy, Steven Jacobson, Genoveffa Franchini, Graham P Taylor, Barrie Hanchard, Owen Morgan, Michael Lairmore

**Affiliations:** 1 Laboratory Medicine and Epidemiology/Biostatistics, University of California at San Francisco and Blood Systems Research Institute, San Francisco, California, USA; 2 Viral Immunology Section, National Institute of Immunology and Neurological Diseases, National Institutes of Health, Bethesda, Maryland, USA; 3 Animal Models & Retroviral Vaccines Section, National Cancer Institute, Bethesda, Maryland, USA; 4 Gastrointestinal and Urogenital Medicine and Communicable Diseases, Imperial College, Norfolk Place, London, United Kingdom; 5 Department of Pathology and Medical Sciences, University of the West Indies, Kingston, Jamaica, West Indies; 6 Center for Retrovirus Research, Department of Veterinary Biosciences, and Comprehensive Cancer Center, The Ohio State University, Columbus, Ohio, USA

## Abstract

Human T-lymphotropic virus type 1 (HTLV-1) and HTLV-2 were among the first human retroviruses discovered in the early 1980's. The International Retrovirology Association is an organized effort that fostered the efforts of scientists and clinicians to form interdisciplinary groups to study this group of retroviruses and their related diseases. The Association promotes excellent science, patient education, and fosters the training of young scientists to promote "bench-to-bedside" research. The *International Conference on Human Retrovirology: HTLV and Related Viruses *sponsored by the Association supports clinicians and researchers in the exchange of research findings and stimulation of new research directions. This years conference will be held from June 22 to 25, in Montego Bay, Jamaica . Since its inception in 1988, these conferences have provided a highly interactive forum for the global community of HTLV scientists. This is of particular importance as HTLV research enters its third decade and a new generation of scientists takes over this important work. Many of the scientists attending the meeting will be from developing countries where HTLV is endemic, consistent with the history of international collaborations that have characterized HTLV research. The International Conference on Human Retrovirology provides a unique opportunity for researchers of all disciplines interested in HTLV infections to meet their peers and to address the questions facing clinicians and scientists who study retroviruses, like HTLV.

## Background

### The International Retrovirology Association: Shared Vision and Common Goals

Albert Einstein once said, "The problems that exist in the world today cannot be solved by the level of thinking that created them". This belief was part of the foundation of The International Retrovirology Association when it was established in May 1994. At that time, informal discussions among established human T-lymphotropic viruses (HTLV) scientists, representing such diverse disciplines as epidemiology, virology, immunology, and clinical medicine, came together at the 6^th ^International HTLV Conference in Absecon, New Jersey, USA. This organized effort from its beginning fostered the efforts of scientists and clinicians to form interdisciplinary groups to study HTLV and its related diseases in a cooperative and innovative manner. With the growth of the conference over the next decade (Table 1), the founders recognized that a professional association was needed to promote shared goals of member scientists and to provide governance and continuity to the international conference and other activities.

**Table 1:** History of The International Conference on Human Retroviruses

**Conference**	**Month/Year**	**Venue**	**Attendance**	**Host**
I	February/1988	Honolulu, Hawaii, USA	100	Dr. Diwan, University of Hawaii, USA
II	March/1989	Port of Spain, Trinidad	114	University of West Indies, Trinidad and Tobago
III	February/1990	Maui, Hawaii, USA	175	Dr. Diwan, University of Hawaii, USA
IV	February/1991	Montego Bay, Jamaica	229	University of West Indies, Jamaica
V	May/1992	Kumamoto, Japan	300	Kumamoto University, Japan
VI	May/1994	Absecon, New Jersey, USA	375	Dr. Stanley Weiss, University Medicine and Dentistry of New Jersey, Absecon, New Jersey, USA
VII	October/1995	Paris, France	320	Drs. De The & A. Gessain, Institute Pasteur, Paris, France
VIII	June/1997	Rio de Janeiro	344	Dr. Pombo de Oliveira, Instituto Nacional de Cancer, Brazil
IX	April/1999	Kagoshima, Japan	366	Profs Osame and Sonoda, Kagoshima University, Japan
X	June/2001	Dublin, Ireland	330	Prof. Hall, University College, Dublin, Ireland
XI	June/2003	San Francisco, California	275	Prof. Murphy, University of California, San Francisco, USA
XII	June/2005	Montego Bay, Jamaica	240*	Prof. Hanchard & Prof. Owen Morgan, University of West Indies, Jamaica

* Abstracts submitted in early registration

The Association has evolved since these humble beginnings to now promote research and education in the field of human retrovirology at the international level, including scientific conferences, interdisciplinary research collaborations, and educational exchanges related to the study of HTLV and related viruses. The Association has chosen to focus on HTLV and other related human and nonhuman primate retroviruses, in part, because numerous other organizations and conferences already exist to study human immunodeficiency virus (HIV). It strives to promote excellent science in the field of HTLV and related viruses and to facilitate the communication of scientific results. The Association fosters the education and training of young scientists who will contribute to and expand the field. It promotes "bench-to-bedside" research that translates findings from the laboratory into clinical trials that benefit HTLV-infected patients. Finally, it promotes awareness of and education about HTLV and related viruses to non-specialist physicians and the broader public.

## Discussion

### HTLVs, Related Retroviruses and Disease Associations

Human T-lymphotropic virus type 1 (HTLV-1) and the closely related HTLV-2 were among the first human retroviruses discovered in the early 1980's[1]. Both viruses are highly related to simian T- lymphotropic viruses (STLV-1 and STLV-2, respectively), presumably from cross-species transmissions of the simian viruses to humans. A recent report of the discovery of two potentially novel, but related HTLVs, indicate that cross species transmission may still occur in situations where humans are exposed to nonhuman primate blood[2]. Thus, in this context the HTLVs are actually members of a broader group of primate T-lymphotropic viruses found worldwide. HTLV-1, is classified as a member of the deltaretrovirus genera, and infects approximately 15 to 20 million people around the world[3]. HTLV-1 causes adult T cell leukemia/lymphoma (ATLL), an aggressive malignancy of CD4+ T lymphocytes in 1 to 5% of infected individuals and comes in a variety of clinical presentations, but is refractory to most forms of therapy[4]. The virus is also associated with a progressive neurologic disease termed HTLV-1-associated myelopathy/tropical spastic paraparesis (HAM/TSP) that affects approximately the same number of infected subjects, but rarely concurrent with ATLL[5]. HTLV-2 does not appear to cause lymphoma or other hematological malignancy, but has been associated with neurologic disease in a small number of infected subjects, and may increase the susceptibility to bacterial infections[6]. HTLV-1 is endemic in Central Africa, the Caribbean, and South America likely due to the slave trade, and southwestern Japan, while HTLV-2 is endemic among Indian tribes of South, Central, and North America. Both viruses may be transmitted from mother to child mostly by breastfeeding, by sexual intercourse, and by blood transfusion and the sharing of contaminated injection apparatus. Injection drug use, with secondary sexual transmission, has resulted in the spread of both HTLV-1 and HTLV-2 in the United States and Europe. The potential contamination of HTLVs in the blood supply makes them an important public health issue in areas with high prevalence and has led many countries including the United States and Japan to screen normal blood donors for these viruses [7-9]. In addition, the HTLVs serve as models for the epidemiology and pathogenesis of other human retroviral infections such as HIV.

### An International Association to Promote Scientific Exchange and Discovery

The International Retrovirology Association accomplishes its goals through a variety of activities. Principally among these is the sponsorship of its biennial general scientific conferences held at rotating international venues, generally in areas with endemic HTLV infection. This unique meeting brings together basic scientists, epidemiologists and clinical researchers in a free form exchange of data to discuss approaches to prevent HTLV infection or develop new therapies against HTLV-mediated diseases. The Association also sponsors smaller symposia and regional meetings directed at specific topics such as disease pathogenesis, treatment of HTLV diseases and regional epidemiology of this group of retroviruses. The group also honors the contributions of leading scientists through endowed awards to leaders in the HTLV research field and promotes partnerships with professional journals to promote the publication of HTLV research and conference proceedings. By funding travel scholarships for the biennial conference to young investigators the association encourages the next generation of retrovirologists and physician-scientists.

#### The International Conference on Human Retrovirology

*HTLV and Related Viruses *is a biennial conference that fulfills the continuing scientific need for the exchange of research findings and stimulation of new research directions. Basic scientists who study the molecular biology of HTLVs continue to grapple with the problem of how these viruses cause cancer. They have discovered important clues in this process by studying the viral gene product called Tax, and other factors that support virus replication. The conference also brings together those scientists that seek to understand the pathogenesis of HAM/TSP, which occurs presumably via aberrant immunologic response to the viral infection. The immune-mediated nature of HAM/TSP in infected subjects with particular HLA genotypes suggest important host factors in understanding this disease and provides a comparative model for other neuro-immunologic diseases such as multiple sclerosis. Clinicians and other scientists employing traditional and molecular epidemiologic tools have been reasonably successful at defining important public health issues related to HTLV infections. These discoveries have led to improved preventative measures to block mother to child transmission, better confirmatory test strategies for blood donor screening, and the prevention of HTLV-2 infection among injection drug users[3]. Despite these advances ongoing clinical and basic research is needed into potential HTLV-1 vaccines, and for improved treatments for ATLL and HAM/TSP. The biennial HTLV conference serves as an important stimulus for all of these research areas.

## Conclusion

### Jamaica Welcomes the International HTLV Conference in 2005

**Figure 1:**
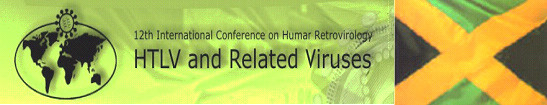
Banner of the *12*^*th *^*International Conference on Human Retroviruses: HTLV and Related Viruses *meeting to be held June 22 to 25, 2005 in Montego Bay, Jamaica.

From June 22^nd ^to 25^th^, 2005, the *12*^*th *^*International Conference on Human Retroviruses: HTLV and Related Viruses *meeting will be held in Montego Bay, Jamaica (Fig. 1). Since its inception in 1988, these conferences have provided a highly interactive forum where the global community of HTLV researchers presents their data at the only meeting devoted exclusively to HTLV and related viruses. This is of particular importance as HTLV research enters its third decade and a new generation of scientists takes over the work of those who started in this field. Many of the scientists attending the meeting will be from developing countries where HTLV is endemic, which is consistent with the history of international collaborations that have characterized HTLV research. Three hundred to 350 scholars from HTLV-endemic regions and infectious disease research institutes gather at this biennial meeting to present their latest data on the molecular virology, immunology, epidemiology and clinical outcomes of HTLV infection.

This year it is appropriate that the conference is held in context to the 25^th ^anniversary of the discovery of the first identified human retrovirus, HTLV-1. HTLV-1 and HTLV-2 infect a wide range of cells in cell culture. Recent reports that indicate that Glut1, the major vertebrate glucose transporter, acts as a HTLV receptor provides new and exciting directions in research of the pathogenesis and anti-viral therapy against HTLV-1[10,11]. Despite recent advances in the management of lymphoproliferative diseases ATLL remains difficult to treat with a median survival of 6 - 9 months. Better treatment is also needed for the chronic and degenerative disorder now known as HAM/TSP[12]. Although the molecular events of virus replication are beginning to be unraveled and knowledge about HTLV-1- and HTLV-2-associated diseases has increased, many questions regarding the pathogenesis of neurologic diseases associated with these retroviruses remain. For example, it is unclear why some HTLV-1-infected subjects develop ATLL or HAM/TSP, whereas the majority of infected individuals remain disease free. More puzzling is the role of these viruses in a number of other inflammatory diseases associated with the HTLV-1 infection including uveitis, thyroiditis, polymyositis, alveolitis, and infective dermatitis. Open questions remain about how HTLV-1 targets and transforms CD4+ lymphocytes and why HTLV-2, while apparently transforming for CD8+ T-lymphocytes in culture is not clearly associated with the devastating clinical disease linked to HTLV-1. Data continue to emerge linking HTLV-1 infection with some impairment of immune function that manifests as a reduced ability to clear, despite therapy infections, certain infections such as Strongyloides stercoralis, Schistosomiasis species, and Sarcoptes scabiei. Recent studies have provided important new roles for the non-structural viral proteins of HTLVs e.g., p12^I^, p13^II^, and p30^II^, which continue to provide important clues into virus replication and T-lymphocyte activation [13-15]. The continued discovery of new, but related members of the deltaretrovirus family of viruses raised intriguing questions regarding the origin and transmission of human retroviruses.

The International Retrovirology Association through its many varied approaches, including its international conference, aims to encourage research in HTLV infections and disease, foster collaborations between research groups, provide a platform for critical analysis of new data and contribute to the dissemination of knowledge about these infections. While the biannual scientific meeting has been the cornerstone of the Association's activities there is increasing recognition of the need for more rapid progress in improving the management of HTLV-associated malignant and inflammatory diseases. The Association is ideally positioned to facilitate this process through its membership and the inclusion of a workshop on clinical trials in ATLL and HAM/TSP in the program of the 12^th ^International Conference on Human Retrovirology. Partnership between clinicians and scientists is key not only to the development of clinical trials but also to pathogenesis studies, which will inform the development of novel interventions. The biannual conference provides a unique opportunity for researchers of all disciplines interested in HTLV infections to meet their peers and truly look beyond their fields to elevate their "level of thinking" to address the questions facing clinicians and scientists who study retroviruses, like HTLV.

## List of Abbreviations

HTLV, human T-lymphotropic viruses

HTLV-1, human T-lymphotropic virus type 1

HTLV-2, human T-lymphotropic virus type 2

HAM/TSP, HTLV-1-associated myelopathy/tropical spastic paraparesis

ATLL, adult T-cell lymphoma/leukemia

## Competing interests

The authors have no competing financial or other interests involved in the data, methods, or writing of this manuscript.

## Authors' contributions

Edward Murphy, Steven Jacobson, Genoveffa Franchini, Graham P. Taylor, Barrie Hanchard, Owen Morgan and Michael D. Lairmore have all met the definition of author as outlined by the Retrovirology journal. Each has made substantive intellectual contributions to the commentary. Each author has given final approval of the version to be published. Each author have participated sufficiently in the work to take public responsibility for appropriate portions of the content.
